# Visualisation of research hotspots in the surgical management of inflammatory bowel disease based on the web of science database

**DOI:** 10.3389/fsurg.2025.1614750

**Published:** 2025-08-06

**Authors:** Xueyi Xue, Baodong Liao, Hao Zeng, Guihe Lin, Dongbo Xu, Shuangming Lin

**Affiliations:** ^1^ Department of Gastroenterology and Anorectal Surgery, Longyan First Hospital, Fujian Medical University, Longyan, China; ^2^ Fujian Medical University, Fuzhou, China

**Keywords:** inflammatory bowel disease, surgical treatment, bibliometrics, VOSviewer, frontiers

## Abstract

**Objective:**

In recent years, there has been a continuous growth in the number of publications related to surgical treatment of IBD globally. However, there is currently a scarcity of bibliometric analyses based on VOSviewer to evaluate the past and present global research in this field. This study aims to analyze the bibliometric characteristics of papers related to IBD surgery to reveal research hotspots and trends in this domain.

**Methods:**

As of August 31, 2024, we retrospectively collected scientific papers on IBD surgery published in the Web of Science Core Collection. Bibliometric metadata from each selected paper was extracted for analysis. VOSviewer was utilized to visualize the results.

**Results:**

A total of 6,239 papers met the inclusion criteria. The United States exhibited the highest total link strength and published the most papers (*n* = 2,334). The University of Toronto's Temerty Faculty of Medicine was the most prolific institution (*n* = 102), while Professor Shen Bo authored the most papers (*n* = 120). The journal “INFLAMMATORY BOWEL DISEASES” published the highest number of relevant papers (*n* = 741). Based on co-occurrence data, keywords were categorized into five clusters, with Cluster 1 and Cluster 2 containing the most prominent keywords.

**Conclusion:**

In this study, a bibliometric analysis of IBD surgery research was conducted using VOSviewer. USA emerged as the leading country in this field and “INFLAMMATORY BOWEL DISEASES” is the most influential journal in this field. Scientists and research institutes all over the world should transcend national boundaries and establish deeper collaboration. The main focus of the research is on the use of robotic surgery in the treatment of IBD, which is essential to expand our understanding of surgical treatment of IBD and to optimise treatment outcomes. Further research in this area could greatly improve the effectiveness of personalised therapies.

## 1 Introduction

Inflammatory bowel disease (IBD) is a group of chronic non-specific inflammatory conditions affecting the gastrointestinal tract with unknown etiologies, encompassing Crohn's disease (CD) and ulcerative colitis (UC). Over the past decades, the incidence of CD has been escalating globally, posing a significant socioeconomic burden (1, 2). Despite advancements in basic and clinical research, the pathophysiological mechanisms underlying IBD remain elusive. Evidence suggests that various factors, including genetics, environment, and microbiota, contribute to the impairment of intestinal epithelial barrier function and immune dysfunction (3, 4).

With the development and application of drugs, particularly biologics, most IBD patients can now achieve remission of intestinal inflammation and mucosal healing through medical therapy. However, nearly half of the patients using immunosuppressants do not experience effective improvement in clinical symptoms, and some patients suffer from significant reductions in quality of life due to drug side effects, incurring substantial healthcare costs (5). Recently, the emergence of biologics such as ustekinumab and vedolizumab has led to a notable decrease in the incidence of side effects, but a proportion of patients still do not respond to this treatment (6, 7). Furthermore, a considerable number of patients require surgical intervention due to complications or failure of medical therapy. Surgical treatment is an integral part of the management system for inflammatory bowel disease. The quality and efficacy of surgery not only determine whether the disease itself can be alleviated or cured, but also significantly impact the long-term quality of life of each IBD patient, reflecting the overall level of diagnosis and treatment for this disease in the current medical landscape.

Bibliometrics, a research field applying quantitative and statistical methods to analyze the production and dissemination of scientific literature, encompasses the collection, organization, and analysis of bibliographic data such as citation counts, collaboration patterns, and publication outputs. The significance of bibliometric indicators, including Impact Factor, CiteScore, Eigenfactor Score, SCImago Journal Rank, and H-index, has grown increasingly prominent with the continuous emergence of scientific discoveries and the widening readership and citation of published research (8). Besides its extensive applications in physics, chemistry, computer science, and other fields, bibliometric analysis offers new research perspectives to the medical domain (9–14). Through analyzing bibliometric indicators, scholars can evaluate the influence of publications, countries, institutions, authors, and journals in specific areas. Furthermore, a significant advantage of bibliometric analysis lies in its ability to reveal dynamic developments and emerging trends in the field by aggregating vast amounts of data (15). Bibliometrics can also identify research trends, emerging areas, and collaborative relationships, providing valuable references for strategic planning and resource allocation in research institutions (16, 17). With the rapid growth of scientific literature and the increasing importance of research impact, bibliometrics will play an increasingly crucial role in research evaluation and assessment.

The number of papers on the surgical management of IBD has grown exponentially over the last two decades. However, there is no VOSviewer-based bibliometric analysis to assess the history and current status of global research in this field. Therefore, the aim of this study was to identify papers on the surgical treatment of IBD, analyse their bibliometric characteristics, and synthesise current findings and trends in order to fill the gap in the literature, reveal the research hotspots and trends in the field, and provide researchers, clinicians, and policy makers with a comprehensive overview of the knowledge and current state of the art in the surgical treatment of IBD.

## 2 Materials and methods

### 2.1 Data source and search strategy

Utilizing Web of Science (WoS) as the primary database for its comprehensiveness and reliability in bibliometric analysis (6), covering over 12,000 academic journals, we conducted a search on August 31st, 2024. The search strategy, agreed upon by all authors and a senior literature search expert, focused on surgical treatments for Inflammatory Bowel Diseases (IBD), Crohn's disease, and Ulcerative colitis. The specific search terms included various combinations of “operative treatment”, “surgical treatment”, “surgery”, “operation”, and related phrases. For further analysis, only English-language articles, including original articles and reviews, were included. Full records and cited references were extracted for further study. Additionally, we examined the top ten most frequently published journals, research directions, and publication years for IBD surgical treatment articles. This study did not require ethical approval as it involved a retrospective bibliometric analysis of published articles.

### 2.2 Data export and analysis

The literature was exported in a “download_*” plain text format and analyzed using Citespace 6.2.R2 and Excel software. This analysis focused on publication volume, countries/research institutions, cited references, and keyword visualization.

### 2.3 Data visualization maps

VOSviewer, a software for bibliometric visualization and analysis (18), was employed to construct visualization maps based on co-authorship data, including authors, countries/regions, organizations, and keyword co-occurrence (19, 20). In these maps, nodes represent specific terms such as keywords, authors, organizations, and countries/regions. The basic parameter settings in VOSviewer are as follows: Method is set to Association strength, Attraction to 2, Rejection to 0, Resolution to 1, and Minimum cluster size to 1. Within the network visualization map based on co-authorship data, the size of nodes indicates the frequency of co-authorship, while lines between two nodes represent collaborative relationships. The thickness of these lines corresponds to Line Strength (LS), which varies based on the number of co-authored papers. Stronger collaborations are denoted by thicker lines, and nodes with high-level collaboration are shown in the same color. The Total Link Strength (TILS) of a term, calculated as the sum of all its links, demonstrates its collaboration intensity with other terms. Keyword visualization maps based on co-occurrence data include three types: network, density, and overlay, each with distinct meanings. In the network visualization map, node size represents the frequency of occurrence, with larger nodes indicating higher frequency and smaller nodes indicating lower frequency. Keywords of the same color form a cluster, and each cluster represents a research hotspot. The keyword network visualization map allows for the identification of research hotspots represented by each cluster (21). In the density visualization map, keyword colors are determined by their frequency of occurrence, enabling the identification of key research foci in the field. By combining this information, one can gain insights into the global research landscape and predict future trends in the field. We selected VOSviewer due to its advantages in visualizing and analyzing bibliometric data. It provides clear and intuitive mapping of citation patterns, which helps in identifying key clusters and trends in the field. Additionally, VOSviewer offers flexible customization options for visualizations, ensuring that the results can be effectively interpreted and presented.

## 3 Results

**Figure 1:**
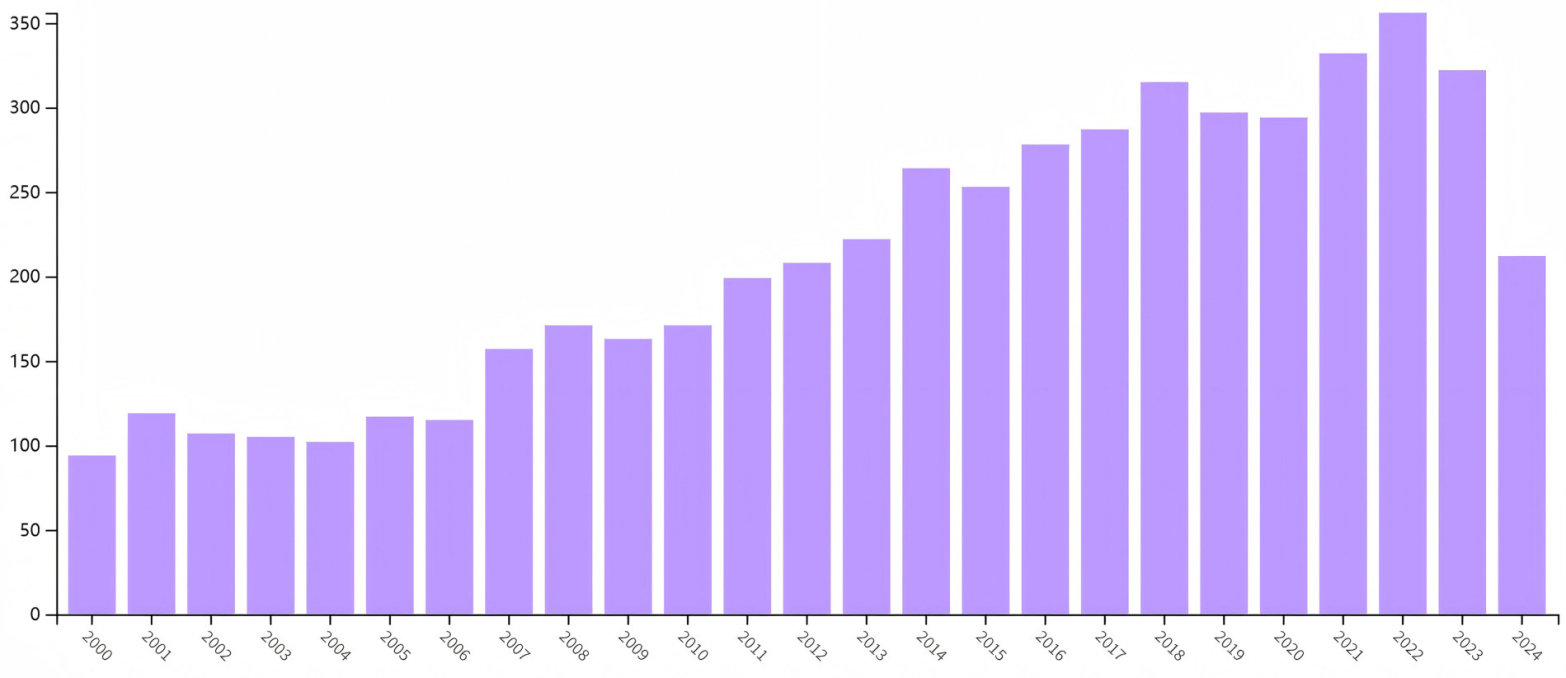
Number of papers published per year from 2000 to 2024.

A total of 6,239 papers were retrieved from WoSCC using specific search criteria and limitations. The Figure 1 illustrates the annual publication count over the past 25 years. In terms of publication type, the majority were original research articles (*n* = 5,340, accounting for 85.591%), while the remainder were review articles (*n* = 899, 14.409%). The line chart reveals a fluctuating yet overall increasing trend in publications between 2000 and 2024, with a peak of 356 articles in 2022. Based on this trend, it is anticipated that the volume of research publications on IBD surgical treatment will continue to rise in the future.

### 3.1 Countries/regions

**Figure 2:**
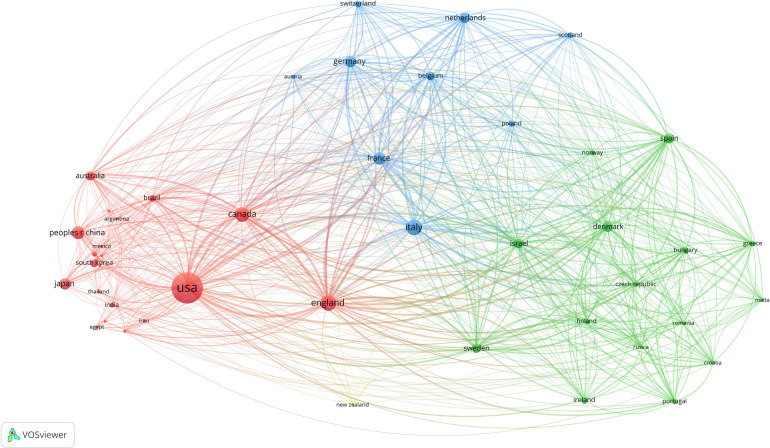
Network visualization map of countries/regions’ co-authorship analysis.

The papers originated from 95 countries/regions, with the top 10 listed in Table 1. The United States had the highest number of publications (*n* = 2,334, accounting for 37.41%), followed by the United Kingdom (*n* = 692, 11.092%), Italy (*n* = 551, 8.832%), Canada (*n* = 427, 6.844%), and Japan (*n* = 375). We visualized the collaborations among countries/regions in the Figure 2. After setting the minimum publication threshold to 10, 46 countries/regions were included. The United States had the highest level of collaboration (TLS = 730), followed by the United Kingdom (TLS = 725) and Italy (TLS = 641). The closest collaborations were between Italy and the United States (LS = 61) and between the United Kingdom and the United States (LS = 548). Evidently, the United States has conducted more research on IBD surgical treatments and engaged in more intensive international collaborations.

**Table 1:** The top 10 authors/organizations/countries ranked by the number of papers.

Countries/regions		Authors		Organizations	
Name	Number of papers	Name	Number of papers	Name	Number of papers
USA	2,334	Shen B	120	University Of Toronto Temerty Faculty Of Medicine	102
ENGLAND	692	Fazio VW	93	University Of Chicago Division Of The Biological Sciences	67
ITALY	551	Remzi FH	90	Icahn School Of Medicine At Mount Sinai Department Of Medicine	62
CANADA	427	Peyrin-biroulet L	79	Mount Sinai Hospital	58
JAPAN	375	Colombel JF	67	Massachusetts General Hospital Division Of Gastroenterology	56
GERMANY	306	Ananthakrishnan AN	65	University Of Toronto Department Of Surgery	56
FRANCE	305	Sandborn WJ	62	Columbia Medical Center	53
PEOPLES R CHINA	293	Lightner AL	60	Imperial College London Faculty Of Medicine	53
SWEDEN	255	Pemberton JH	60	University Of Calgary Cumming School Of Medicine	50
NETHERLANDS	240	Loftus EV	55	Icahn School Of Medicine At Mount Sinai Division Of Gastroenterology	49

### 3.2 Institutional and individual author analysis

**Figure 3:**
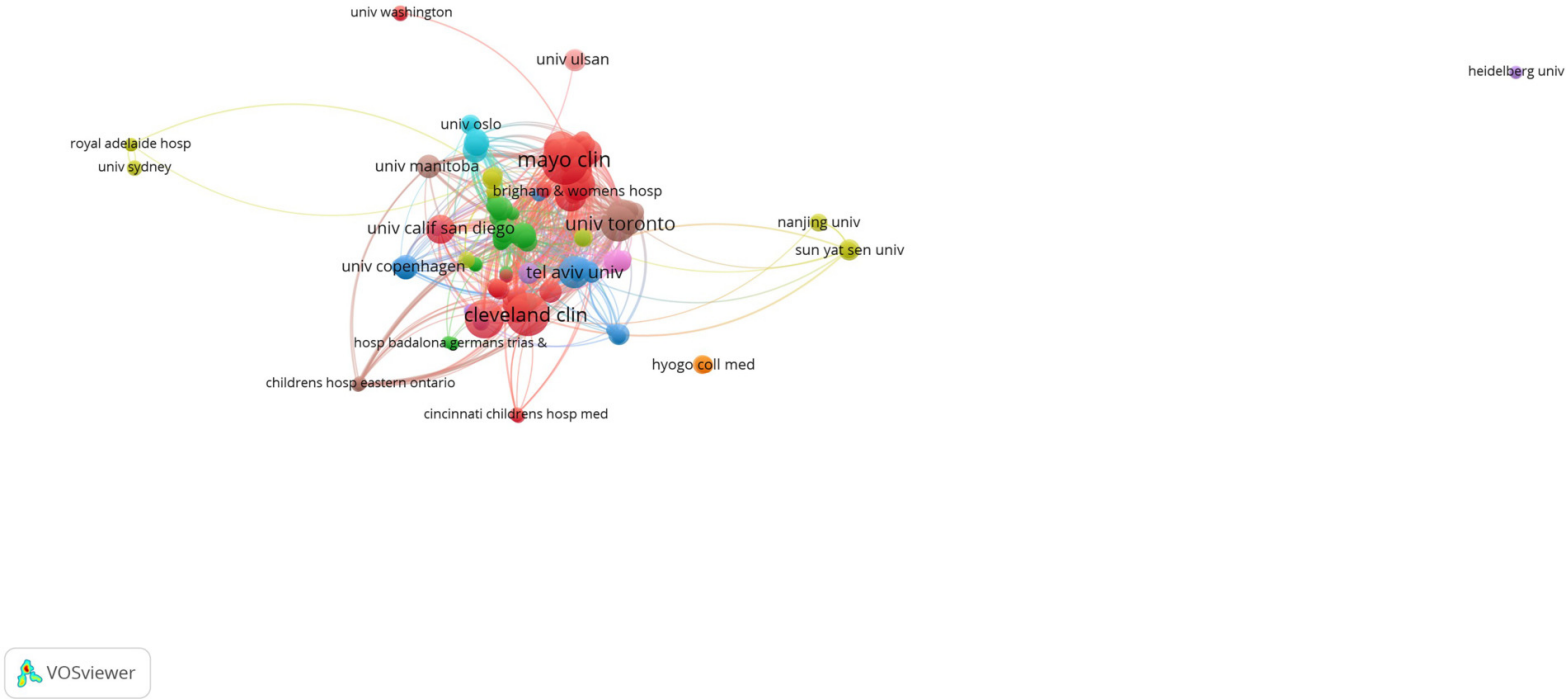
Network visualization map of institutional analysis.

To explore the contributions of various institutions to IBD surgical research, we analyzed their publication output. Globally, approximately 4,190 institutions with a total of 22,602 researchers have contributed to studies on IBD surgeries. Table 1 lists the top 10 research institutions and their leading investigators. The University of Toronto's Temerty Faculty of Medicine stands out with the highest number of publications (*n* = 102), followed by the University of Chicago Division of the Biological Sciences (*n* = 67) and another institution (note: the third institution in the original text appears to be a repetition and should be replaced with the next highest-ranking institution, details not provided in the original text) (*n* = 61). Among the top 10 institutions, 6 are from the United States, 3 from Italy, and 1 from the United Kingdom. Based on co-authorship data, we constructed a network visualization map of these institutions (Figure 3). For the collaborative analysis, a minimum publication threshold of 10 papers was set. A total of 212 institutions met this criterion and were included in the co-authorship analysis. The institution with the highest Total Link Strength (TLS) is the University of Toronto's Temerty Faculty of Medicine (TLS = 303), followed by the University of Calgary's Cumming School of Medicine (TLS = 207). The strongest collaboration was observed between the University of Toronto's Temerty Faculty of Medicine and Mount Sinai Hospital (LS = 23). Notably, the University of Toronto's Temerty Faculty of Medicine, along with several other Canadian and American institutions, made significant contributions to the early development of IBD surgeries.

**Figure 4:**
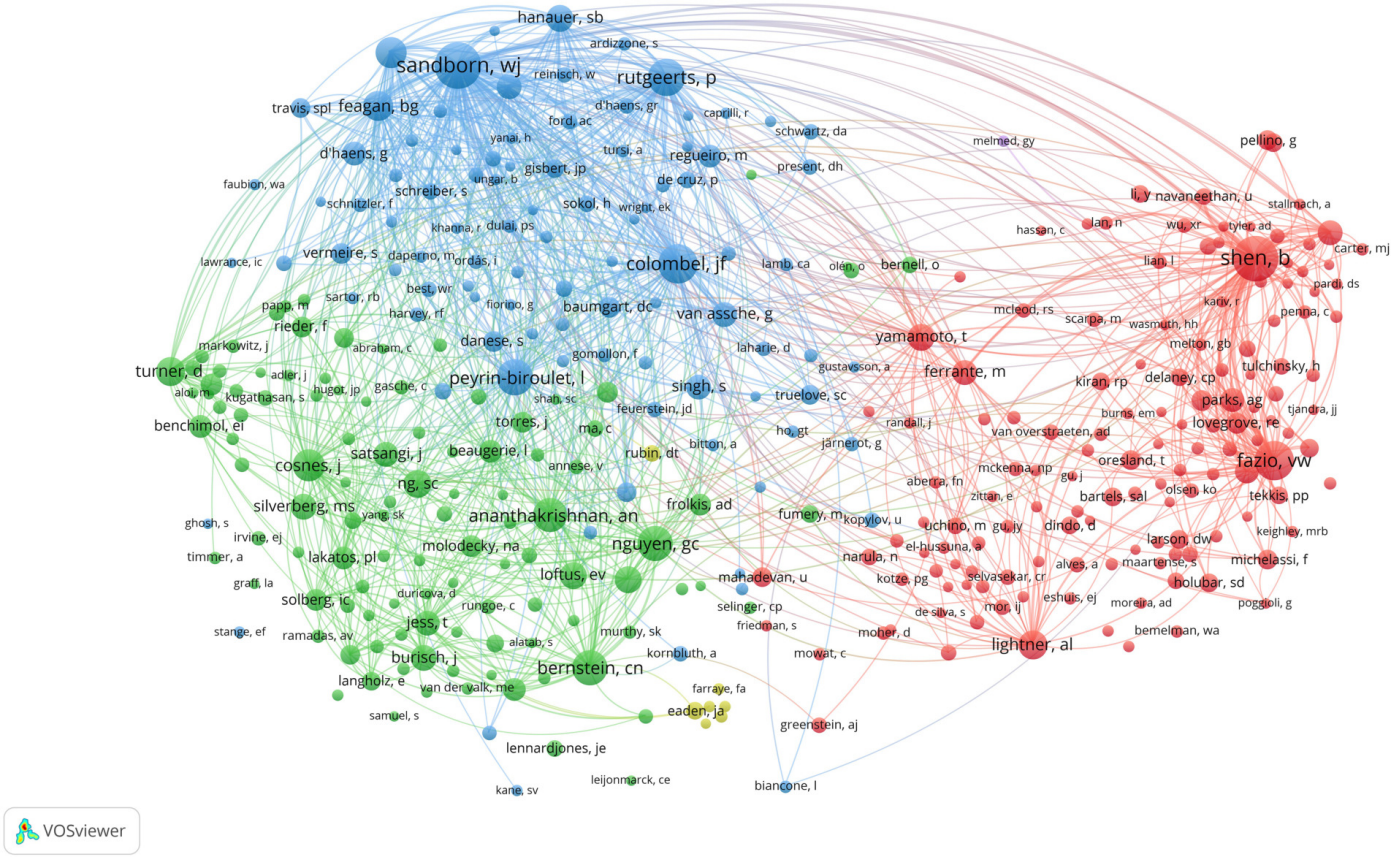
Network visualization map of authors’ co-authorship analysis.

In terms of individual authors, Shen Bo has the highest number of publications (*n* = 120), followed by Fazio, Victor W. (*n* = 93), and Remzi, Feza H. (*n* = 90). Utilizing co-authorship data, we generated a network visualization of author collaborations, as shown in Figure 4. For this analysis, a minimum of 10 publications was required. A total of 198 authors qualified and were included. Professor Peyrin-Biroulet Laurent leads with the highest TLS (TLS = 139), closely followed by Professor Shen Bo (TLS = 137) and Professor Remzi, Feza H. (TLS = 134). The closest collaboration was observed between Professor Shen Bo and Professor Remzi, Feza H (LS = 26).

### 3.3 Citations and journals

**Figure 5:**
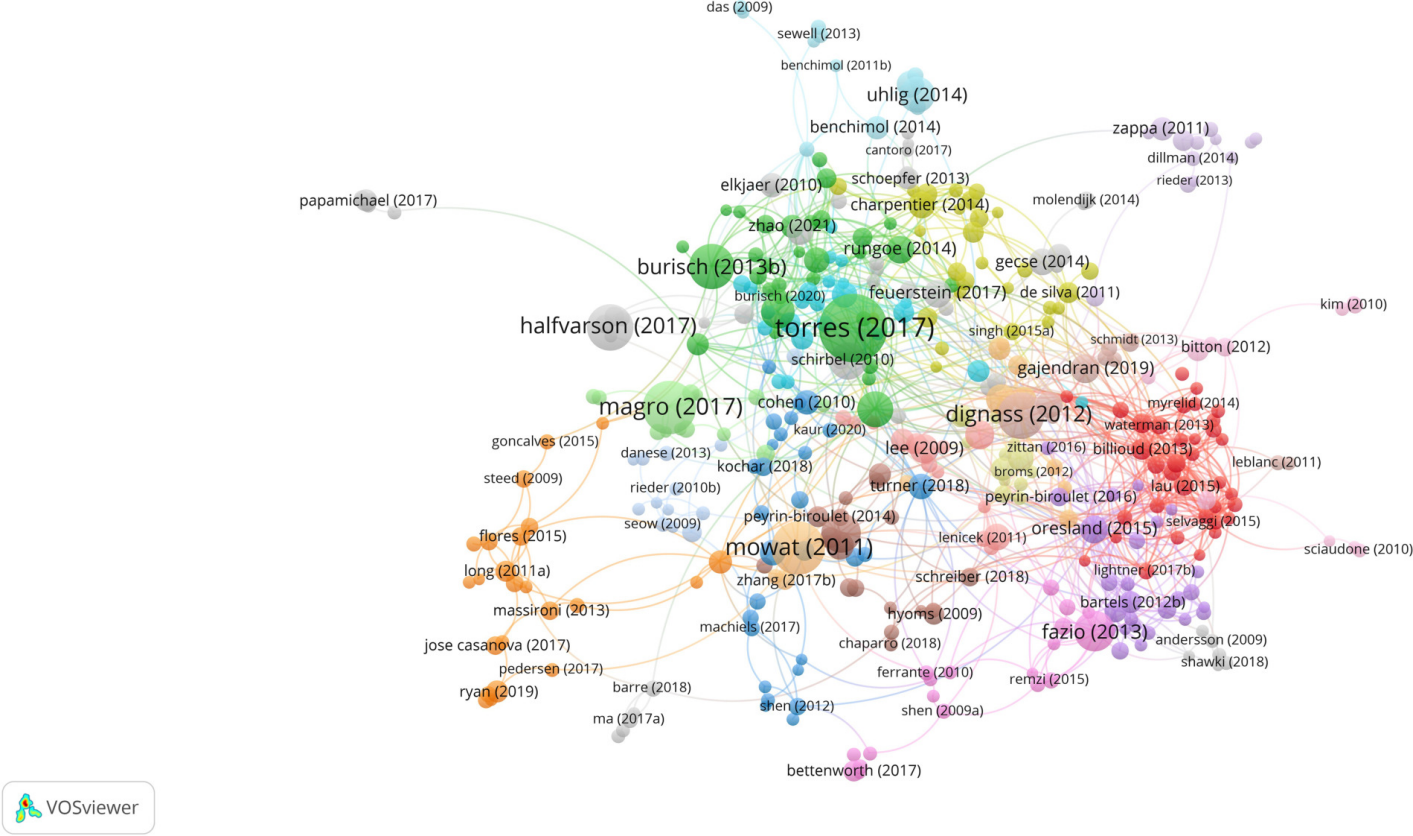
Network visualization map of citing articles analysis.

The frequency of citations can be used to evaluate the most highly cited articles in this field. Table 2 lists the top 5 most cited articles, all of which have been cited over 300 times. The article “Third European Evidence-based Consensus on Diagnosis and Management of Ulcerative Colitis. Part 1: Definitions, Diagnosis, Extra-intestinal Manifestations, Pregnancy, Cancer Surveillance, Surgery, and Ileo-anal Pouch Disorders (Publication with Expression of Concern)”, published in the Journal of Crohn's & Colitis in 2017, is the most frequently cited work, providing significant evidence for the advancement of IBD surgery. We have constructed a visual network diagram of citing articles (Figure 5), setting the minimum citation count at 50. A total of 397 articles meet this criterion, with most of them published in the past 10 to 20 years. These highly cited articles have become a crucial knowledge foundation for the development of IBD surgery. This study encompassed 1,222 articles published in 405 journals. The Table 3 presents the top 10 journals by publication volume and their latest Impact Factors (IF). Among the top 10 journals, 2 are ranked in the first quartile (Q1) of Journal Citation Reports (JCR). Five of these publishers are from the United States, while China, Sweden, the United Kingdom, and Germany each have one represented.

**Table 2:** Top 5 highly cited analyses of inflammatory bowel disease surgery literature.

Article title	Author's name	Citations
Third European Evidence-based Consensus on Diagnosis and Management of Ulcerative Colitis. Part 1: Definitions, Diagnosis, Extra-intestinal Manifestations, Pregnancy, Cancer Surveillance, Surgery, and Ileo-anal Pouch Disorders (Publication with Expression of Concern)	Magro, F	957
ILEAL POUCH-ANAL ANASTOMOSES COMPLICATIONS AND FUNCTION IN 1,005 PATIENTS	FAZIO, VW	955
Risk of Surgery for Inflammatory Bowel Diseases Has Decreased Over Time: A Systematic Review and Meta-analysis of Population-Based Studies	Frolkis, AD	614
Ileal Pouch Anal Anastomosis Analysis of Outcome and Quality of Life in 3,707 Patients	Fazio, VW	576
J ileal pouch-anal anastomosis for chronic ulcerative colitis: complications and long-term outcome in 1,310 patients	Meagher, AP	457

**Table 3:** The top 10 journals ranked by the number of papers.

Journals	Number of papers	IF	Country of journal affiliation
INFLAMMATORY BOWEL DISEASES	741	5.272, Q1	United States of America
DISEASES OF THE COLON RECTUM	407	3.684, Q2	United States of America
JOURNAL OF CROHNS COLITIS	229	4.577, Q2	United States of America
INTERNATIONAL JOURNAL OF COLORECTAL DISEASE	171	2.667, Q3	German
COLORECTAL DISEASE	165	3.251, Q3	England
ALIMENTARY PHARMACOLOGY THERAPEUTICS	155	7.133, Q2	England
SCANDINAVIAN JOURNAL OF GASTROENTEROLOGY	147	2.429, Q3	Sweden
WORLD JOURNAL OF GASTROENTEROLOGY	142	2.848, Q3	China
DIGESTIVE DISEASES AND SCIENCES	132	4.286, Q2	United States of America
AMERICAN JOURNAL OF GASTROENTEROLOGY	125	11.217, Q1	United States of America

### 3.4 Keyword co-occurrence analysis

**Figure 6:**
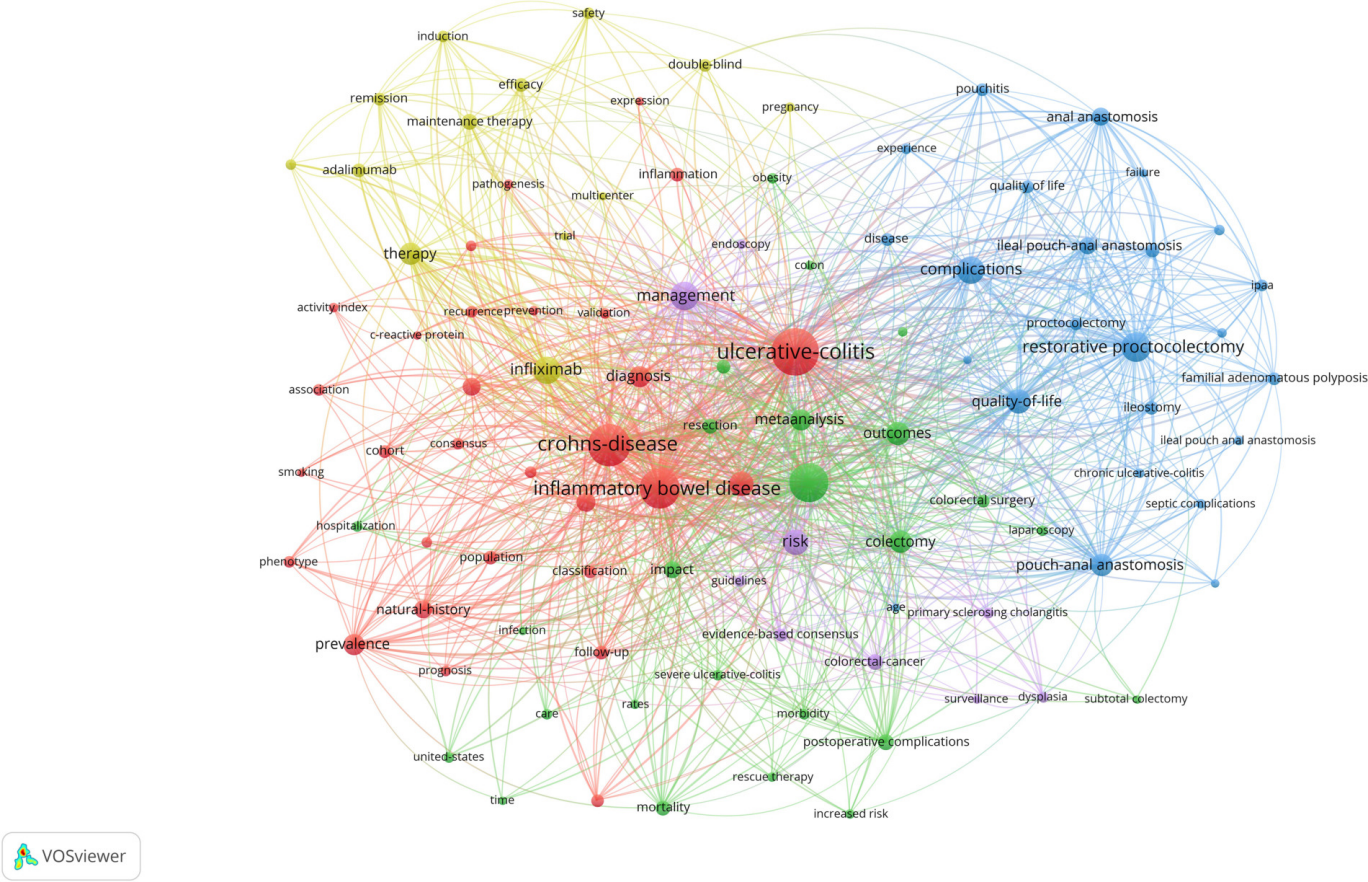
Network visualization map of keyword co-occurrence analysis conducted by VOSviewer.

According to the keyword co-occurrence analysis, VOSviewer identified 8,586 keywords from 6,239 papers through co-occurrence data analysis. After limiting the keyword occurrences to at least 50 times, 115 keywords were finally selected. Following manual unification and standardization, 101 keywords were ultimately determined. Based on these keywords, a network visualization map was constructed and divided into five clusters (Figure 6). Research hotspots were identified based on the keywords within each cluster, as detailed in the Figure 6. Cluster 1, the largest in this study, focuses on activity index, pathogenesis, postoperative recurrence, predictive factors, prognosis, and risk factors. Cluster 2 centers on surgical procedures, inpatient treatment, and laparoscopy. Cluster 3 mainly concerns complications, experience, long-term outcomes, and quality of life. Cluster 4 highlights efficacy, maintenance therapy, safety, treatment, and clinical trials. Frequently occurring keywords in Cluster 5 include colorectal cancer, dysplasia, and primary sclerosing cholangitis.

**Figure 7:**
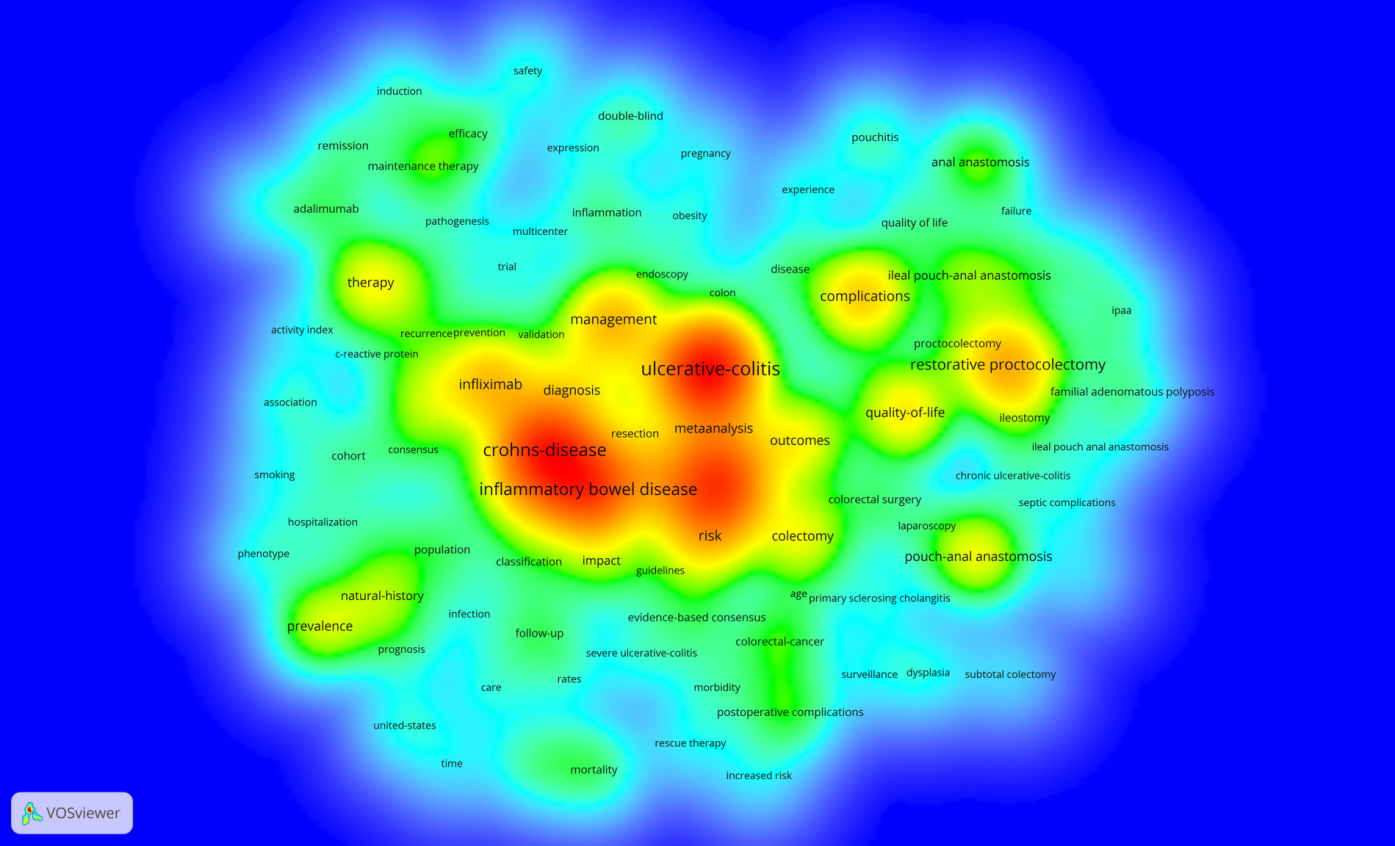
Density visualization map of keyword co-occurrence analysis conducted by VOSviewer.

We constructed a keyword density visualisation map based on the frequency of keywords, as shown in Figure 7. The main keywords included IBD (frequency: 1,002), UC (frequency: 1,393), CD (frequency: 1,131), surgery (frequency: 960) and management (frequency: 513).

## 4 Discussion

### 4.1 Key findings

Utilizing VOSviewer for bibliometric analysis, this study aimed to identify publications on surgical treatments for IBD and analyze their bibliometric characteristics. Through WOSCC, we retrieved 6,239 relevant papers spanning from 1975 to 2024. The number of publications in this field has shown a general increase over the years, indicating a rapid development phase for IBD surgical treatment research. This growth may be attributed to the recognition that, despite the widespread use of biologics in IBD therapy and their effectiveness in symptom relief for some patients, early surgical intervention is still necessary for certain individuals to prevent disease progression and depletion of bodily reserves (22). Consequently, research institutions have continuously strengthened their support for IBD treatment-related studies, with increasing research funding, thereby driving rapid advancements in the field. Notably, the field reached a peak in 2022 with 356 publications, followed by a slight decline in 2023, possibly due to incomplete WOS coverage from 2023 to 2024. The past decade has particularly witnessed a surge in IBD surgery research, with the growth rate of publications far exceeding that of the previous ten years. These findings suggest that this research area is attracting increasing attention from the scientific community.

Based on the visual map of co-authorship data across countries/regions, it can be concluded that despite the global attention garnered by research in this field, the distribution of studies related to IBD surgery remains uneven. The economic environment significantly impacts the level of research and development (23). Consequently, this research area is primarily concentrated in European countries with higher economic levels. The findings reveal that over one-third of the papers were published in the United States, reflecting its dominant position in this field. Similar patterns have been observed in bibliometric analyses of other areas, such as endometrial cancer (24) and urological robotic surgery research (25). This may be attributed to the high level of funding for academic activities and the long history of surgical research in the United States. Furthermore, the United States has the closest collaboration with other countries in this research field, while many other countries only have partnerships with a few nations. Therefore, cooperation among countries should be further strengthened.

Professor Shen Bo from the Key Laboratory of Systems Medicine and Precision Diagnosis and Therapy of Taizhou, Taizhou, China, ranks first in terms of the number of published papers, with a total of 120 IBD-related papers. Among all institutions, the Temerty Faculty of Medicine at the University of Toronto tops the global list, publishing 102 relevant papers, accounting for 1.635% of all papers in this field. The vast majority of the top 10 contributing institutions were from the United States. Collaboration between authors and organisations was also noted. Professor Peyrin-biroulet laurent from the Department of Gastroenterology, Nancy University Hospital, Vandœuvre-lès-Nancy, France. was the author most widely associated with other scientists, while the University Of Toronto Temerty Faculty Of Medicine is the most associated with other institutions in the field. International collaborations centred on the United States are at the forefront of the field. The above findings confirm the key contribution and leadership of the United States in neoadjuvant immunotherapy research, which may stem from its national economic conditions and high level of investment in healthcare. This field would benefit from extensive international collaboration, which would increase the overall level of research. Network visualisation maps of co-authorship analyses show that collaboration between authors is limited to small groups and collaborative links between research institutions are lacking. This phenomenon suggests that scientists and institutions from all over the world should push the boundaries and achieve deep collaboration. Only then can we promote the rapid development of this field for the benefit of patients.

Peer-reviewed journals are crucial for academic publishing. Core journals often publish essential research in the field. Researchers can identify potential journal submissions based on the number of journals publishing in the field of neoadjuvant immunotherapy. INFLAMMATORY BOWEL DISEASES (Frontiers in Oncology) published the most papers with 741. The journal with the highest impact factor among the top 10 published papers was American Journal of Gastroenterology (IF11.217), followed by Alimentary Pharmacology & Therapeutics (IF7.133). Impact factor and JCR are commonly used metrics for evaluating the impact of journals.JCR classifies all journals into four quartiles (Q1–Q4) based on the impact factor. Among the top ten journals in terms of number of papers, Q1 journals account for 20%. In addition, Asian publishers are underrepresented in the top 10 journals, despite the outstanding contribution of Chinese researchers in IBD research. There is a need to establish and develop journals with international impact in Asia. Overall, INFLAMMATORY BOWEL DISEASES, DISEASES OF THE COLON RECTUM, and JOURNAL OF CROHNS COLITIS are the journals with the highest overall comprehensiveness in the field of research, both in terms of impact factor and number of papers published.

This study aims to answer a topical research question about a research that has been extensively studied by researchers over a certain period of time. The number of citations can be used as one of the indicators of the academic impact of a paper. In highly cited publications often represent the underlying theme of a research area. Research hotspots can be identified by counting the number of citations and identifying highly cited publications. For papers, the number of citations can be related to many factors, such as year of publication and accessibility. In terms of year of publication, even the most cited papers were not cited when they were originally published, and older papers may have more citations due to the cumulative effect (26). In this study, the five most frequently cited publications were published between 2021 and 2023 and focused on complications, quality of life, and function in the surgical treatment of IBD.

The keywords in the network visualization map are divided into six clusters. Cluster 1, the largest in this study, focuses on activity index, pathogenesis, postoperative recurrence, predictive factors, prognosis, and risk factors. Cluster 2 centers around surgical procedures, inpatient treatment, and laparoscopy. Cluster 3 highlights complications, experience, long-term outcomes, and quality of life. Cluster 4 emphasizes efficacy, maintenance therapy, safety, treatment, and trials. Keywords such as colorectal cancer, dysplasia, and primary sclerosing cholangitis are frequently used in Cluster 5.

Keywords reflect the core content of research, and co-occurrence analysis can identify high-frequency keywords in different studies, helping researchers quickly grasp research hotspots. The keywords in the network visualization map are divided into six clusters. Cluster 1 focuses on the biological characteristics and clinical progression of IBD. Keywords such as “activity index”, “pathogenesis”, and “postoperative recurrence” suggest that this cluster may involve research on tumor growth, spread, and recurrence mechanisms. “Predictive factors”, “prognosis”, and “risk factors” imply that researchers may be seeking factors that can predict disease progression or patient survival rates. There is a complex relationship between surgical treatment for inflammatory bowel disease (IBD) and its biological characteristics and clinical progression, influenced by multiple factors such as IBD type, disease severity, treatment methods, and patient demographics. The clinical progression of IBD often determines the necessity of surgical intervention, and IBD patients, especially those with Crohn's disease (CD), have a relatively higher surgical rate compared to patients with ulcerative colitis (UC) (27). With the introduction of new drugs such as biologics, IBD treatment methods have changed. Studies have shown that early and continuous use of biologics may reduce the need for surgery in UC patients, but the effect on CD patients is still unclear (28). Thus, the interaction between IBD's biological characteristics and clinical progression significantly impacts treatment strategies and surgical outcomes. A deep understanding of these relationships is crucial for optimizing patient care and improving quality of life.

Cluster 2 focuses on surgical treatments for IBD. With keywords such as “surgical procedures”, “inpatient care”, and “laparoscopy”, the studies explore various surgical techniques, inpatient management, and the application of minimally invasive surgeries like laparoscopy. Significant advancements have been made in IBD surgical treatment, especially in the utilization of minimally invasive surgical (MIS) techniques. Compared to traditional open surgeries, minimally invasive procedures like laparoscopy and robotic-assisted surgeries offer notable benefits, including shorter hospital stays, faster recovery, and reduced postoperative complication rates (29, 30). As an emerging technique, transanal surgery demonstrates distinct advantages in managing low rectal lesions (31). Minimally invasive surgeries are suitable for a range of IBD conditions, including Crohn's disease and ulcerative colitis. They provide comparable long-term disease control to open surgeries but with lower mortality and complication rates (29, 30). Thus, with the continuous evolution of minimally invasive techniques, surgical treatments for IBD have become safer and more effective, bringing greater benefits to patients.

Cluster 3 focuses on the complications after surgical treatment of IBD and the long-term health status of patients, while Cluster 4 emphasizes the efficacy and safety of surgical approaches for IBD. The term “complications” indicates the exploration of potential issues arising from surgical or other therapeutic interventions. “Experience” refers to the expertise of doctors or medical institutions in managing these complications. Additionally, “long-term outcomes” and “quality of life” evaluate the overall health and well-being of patients following treatment. Keywords such as “efficacy”, “maintenance therapy”, “safety”, “treatment”, and “trial” suggest a comparative analysis of various surgical options, assessing their effectiveness and associated risks. For IBD patients who do not respond to medication or develop severe complications, surgery may become a necessary intervention. However, IBD surgeries carry risks of complications, including infection, bleeding, intestinal stenosis, fistula formation, bowel obstruction, and malnutrition (32, 33). Simultaneously, the long-term health of patients, encompassing disease recurrence, cancer risk, quality of life, and mental health, is also a subject of concern (34). Although surgery can provide symptom relief for some, its impact on quality of life varies among individuals (35). Therefore, regular monitoring and appropriate follow-up of surgical patients are crucial for maintaining long-term health (36).

Cluster 5 may be dedicated to specific types of IBD or related conditions, as indicated by keywords such as “colon cancer”, “dysplasia”, and “primary sclerosing cholangitis (PSC)”, suggesting a focus on the characteristics, diagnosis, treatment, or prognosis of these specific conditions. Inflammatory bowel disease (IBD) is a complex chronic condition that can lead to various complications including abnormal bone metabolism, malnutrition, iron deficiency, and immune-related disorders (37–39). These complications reflect the systemic impact of IBD, emphasizing the importance of comprehensive treatment and management. To better control the disease and improve patients' quality of life, both physicians and patients need to closely monitor these potential complications and take corresponding therapeutic measures.

In conclusion, co-occurrence analysis of high-frequency keywords in studies can highlight areas of focus. Expanding our understanding of surgical treatment of IBD is essential to optimise outcomes. Further research in this area could significantly enhance the effectiveness of personalised treatment.

### 4.2 Advantages, limitations, and suggestions for future research

This bibliometric analysis employed VOSviewer to examine IBD surgery-related papers, aiming to understand the global research status and forecast future trends in this field, distinct from traditional literature reviews. The study further assisted researchers in identifying influential authors, organizations, and journals within this domain. Scholars interested in IBD surgery can engage in academic activities or seek collaborations with relevant scholars or institutions. Additionally, our findings can guide researchers in this field to submit their manuscripts to appropriate journals.

However, there are certain limitations to this study. Firstly, it only included articles written in English and recorded in the WoSCC database. Although this may not affect the overall trend of the results as WoSCC covers most high-quality research. Secondly, recently published high-quality studies may not have received due attention due to citation delays, requiring updates in subsequent research. Despite these limitations, this study significantly aids researchers in comprehending the advancements, hotspots, trends, and frontiers of IBD treatment, as well as identifying areas needing further investigation.

### 4.3 Limitation

Our study has several limitations that could influence the generalizability of our findings. First, the exclusive use of English-language publications and the WoSCC database may introduce bias and limit the comprehensiveness of our results. This approach might overlook valuable research conducted in non-English speaking countries, potentially skewing our findings toward perspectives prevalent in the English academic sphere. Additionally, while the WoSCC database is authoritative and widely used, it does not encompass all industry reports and grey literature, which could provide insights into practical applications and emerging trends not yet captured in traditional academic publications. These limitations might affect the applicability of our conclusions across different regions and research environments. In future research, we aim to address these issues by expanding our data collection to include multi—lingual resources and alternative databases, and by incorporating practical surveys to minimize bias and enhance the generalizability of our findings.

## 5 Conclusion

To our knowledge, this is the first bibliometric analysis of IBD surgery research based on VOSviewer. In this field, the United States is the leading country, and “Inflammatory Bowel Diseases” is the journal with the most publications in this area. Scientists and institutions from around the world should break through boundaries and carry out deep cooperation. The main research topics of IBD surgery have always been treatment effects and postoperative complications.

In summary, this study utilizes a bibliometric analysis method based on VOSviewer to elaborate the current research status and development trends of IBD surgery. Simultaneously, this study identifies the most prolific researchers and institutions in this field, aiding scholars in finding suitable research subjects and scientific research partners, and laying the foundation for international cooperative research in this area.

## Data Availability

The original contributions presented in the study are included in the article/Supplementary Material, further inquiries can be directed to the corresponding author.

## References

[1] Ng SC Shi HY Hamidi N Underwood FE Tang W Benchimol EI Worldwide incidence and prevalence of inflammatory bowel disease in the 21st century: a systematic review of population-based studies. Lancet. (2017) 390(10114):2769–78. 10.1016/S0140-6736(17)32448-0 Epub 2017 October 16. Erratum in: *Lancet*. (2020) **396**(10256):e56. doi: 10.1016/S0140-6736(20)32028-6. 29050646

[2] Singh S Qian AS Nguyen NH Ho SKM Luo J Jairath V Trends in U.S. health care spending on inflammatory bowel diseases, 1996–2016. Inflamm Bowel Dis. (2022) 28(3):364–72. 10.1093/ibd/izab074 33988697 PMC8889287

[3] de Souza HSP Fiocchi C Iliopoulos D. The IBD interactome: an integrated view of aetiology, pathogenesis and therapy. Nat Rev Gastroenterol Hepatol. (2017) 14(12):739–49. 10.1038/nrgastro.2017.110 28831186

[4] Li N Shi RH. Updated review on immune factors in pathogenesis of Crohn’s disease. World J Gastroenterol. (2018) 24(1):15–22. 10.3748/wjg.v24.i1.15 29358878 PMC5757119

[5] Hisamatsu T Matsumoto T Watanabe K Nakase H Motoya S Yoshimura N Concerns and Side effects of azathioprine during Adalimumab induction and maintenance therapy for Japanese patients with crohn’s disease: a subanalysis of a prospective randomised clinical trial [DIAMOND study]. J Crohns Colitis. (2019) 13(9):1097–104. 10.1093/ecco-jcc/jjz030 PMID: 30753377. 30753377

[6] Straatmijer T Biemans VBC Hoentjen F de Boer NKH Bodelier AGL Dijkstra G Ustekinuma b for Crohn’s disease: two-year results of the initiative on crohn and colitis (ICC) registry, a nationwide prospective observational cohort study. J Crohns Colitis. (2021) 15(11):1920–30. 10.1093/ecco-jcc/jjab081 33909062

[7] Vermeire S Loftus EV Jr Colombel JF Feagan BG Sandborn WJ Sands BE Long-term efficacy of vedolizumab for Crohn’s disease. J Crohns Colitis. (2017) 11(4):412–24. 10.1093/ecco-jcc/jjw176 27683798

[8] Durieux V Gevenois PA. Bibliometric indicators: quality measurements of scientific publication. Radiology. (2010) 255(2):342–51. 10.1148/radiol.09090626 20413749

[9] Yeung AWK Tosevska A Klager E Eibensteiner F Laxar D Stoyanov J Virtual and augmented reality applications in medicine: analysis of the scientific literature. J Med Internet Res. (2021) 23(2):e25499. 10.2196/25499 33565986 PMC7904394

[10] He X Peng C Xu Y Zhang Y Wang Z. Global scientific research landscape on medical informatics from 2011 to 2020: bibliometric analysis. JMIR Med Inform. (2022) 10(4):e33842. 10.2196/33842 35451986 PMC9073618

[11] Ahmad P Slots J. A bibliometric analysis of periodontology. Periodontol 2000. (2021) 85(1):237–40. 10.1111/prd.12376 33226679

[12] Zheng F Wang L Zeng Z Wu S. International technologies on prevention and treatment of neurological and psychiatric diseases: bibliometric analysis of patents. JMIR Ment Health. (2022) 9(2):e25238. 10.2196/25238 35191849 PMC8905476

[13] Zhu Y Zhang C Wang J Xie Y Wang L Xu F. The top 100 highly cited articles on anterior cruciate ligament from 2000 to 2019: a bibliometric and visualized analysis. Orthop Traumatol Surg Res. (2021) 107(8):102988. 10.1016/j.otsr.2021.102988 34146752

[14] He D Cao S Le Y Wang M Chen Y Qian B. Virtual reality technology in cognitive rehabilitation application: bibliometric analysis. JMIR Serious Games. (2022) 10(4):e38315. 10.2196/38315 36260388 PMC9631168

[15] Roldan-Valadez E Salazar-Ruiz SY Ibarra-Contreras R Rios C. Current concepts on bibliometrics: a brief review about impact factor, Eigenfactor score, CiteScore, SCImago Journal rank, source-normalised impact per paper, H-index, and alternative metrics. Ir J Med Sci. (2019) 188(3):939–51. 10.1007/s11845-018-1936-5 30511320

[16] Zhao X Nan D Chen C Zhang S Che S Kim JH. Bibliometric study on environmental, social, and governance research using CiteSpace. Front Environs Sci. (2023) 10. 10.3389/fenvs.2022.1087493

[17] Mukherjee D Lim WM Kumar S Donthu N. Guidelines for advancing theory and practice through bibliometric research. J Bus Res. (2022) 148:101–15. 10.1016/j.jbusres.2022.04.042

[18] van Eck NJ Waltman L. Software survey: vOSviewer, a computer program for bibliometric mapping. Scientometrics. (2010) 84(2):523–38. 10.1007/s11192-009-0146-3 20585380 PMC2883932

[19] Nan D Sun S Gopi S Lee KM Kim JH. A bibliometric analysis of metaverse research using VOSviewer. 2023 17th International Conference on Ubiquitous Information Management and Communication (IMCOM), Seoul, Korea, Republic of (2023). p. 1–4. 10.1109/IMCOM56909.2023.10035584

[20] Peng C He M Cutrona SL Kiefe CI Liu F Wang Z. Theme trends and knowledge structure on mobile health apps: bibliometric analysis. JMIR Mhealth Uhealth. (2020) 8(7):e18212. 10.2196/18212 32716312 PMC7418015

[21] Rivera-Sotelo N Vargas-Del-Angel RG Ternovoy SK Roldan-Valadez E. Global research trends in COVID-19 with MRI and PET/CT: a scoping review with bibliometric and network analyses. Clin Transl Imaging. (2021) 9(6):625–39. 10.1007/s40336-021-00460-x 34414137 PMC8364406

[22] Gionchetti P Dignass A Danese S Magro Dias FJ Rogler G Lakatos PL 3rd European evidence-based consensus on the diagnosis and management of Crohn’s disease 2016: part 2: surgical management and special situations. J Crohns Colitis. (2017) 11(2):135–49. 10.1093/ecco-jcc/jjw169. Erratum in: *J Crohns Colitis*. (2023) **17**(1):149. doi: 10.1093/ecco-jcc/jjac104. PMID: 27660342. 27660342

[23] Lu H Li Y Chen M Kim H Serikawa S. Brain intelligence: go beyond artificial intelligence. Mobile Netw Appl. (2018) 23:368–75. 10.1007/s11036-017-0932-8

[24] Xiao P Yao C Wang G. The top 100 most cited papers on endometrial carcinoma: a bibliometric analysis. Front Oncol. (2022) 12:987980. 10.3389/fonc.2022.987980 36059668 PMC9433873

[25] Jackson SR Patel MI. Robotic surgery research in urology: a bibliometric analysis of field and top 100 articles. J Endourol. (2019) 33(5):389–95. 10.1089/end.2018.0866 30892070

[26] Ahmad SS Evangelopoulos DS Abbasian M Röder C Kohl S. The hundred most-cited publications in orthopaedic knee research. J Bone Joint Surg Am. (2014) 96(22):e190. 10.2106/JBJS.N.00029 25410518

[27] Chaparro M Garre A Núñez Ortiz A Diz-Lois Palomares MT Rodríguez C Riestra S TIncidence, clinical characteristics and management of inflammatory bowel disease in Spain: large-scale epidemiological study. J Clin Med. (2021) 10(13):2885. 10.3390/jcm10132885. Erratum in: *J Clin Med*. (2022) **11**(19):5816. doi: 10.3390/jcm11195816. 34209680 PMC8268420

[28] Pollok RCG Jayasooriya N. Editorial: early and persistent biological treatment and its impact on long term surgical outcomes in inflammatory bowel disease. Aliment Pharmacol Ther. (2022) 55(3):370–1. 10.1111/apt.16747 35040166

[29] Zaman S Mohamedahmed AYY Yassin NA. Minimally invasive surgery for inflammatory bowel disease: a systematic review and meta-analysis of robotic versus laparoscopic surgical techniques. J Crohns Colitis. (2024) 18(9):1522–3. 10.1093/ecco-jcc/jjae065 38780033

[30] Abad CCU Llumiquinga DBL Imbaquingo HJR Rueda RAL Rueda JEL Moreno JDN The use of minimally invasive surgery in the treatment of Crohn’s disease: a systematic review of evidence. Int J Med Sci Clin Res Stud. (2024) 4(5). 10.47191/ijmscrs/v4-i05-17

[31] Yuen A Brar MS de Buck van Overstraeten A. Indications and surgical technique for transanal proctectomy and ileal pouch-anal anastomosis for inflammatory bowel disease. Clin Colon Rectal Surg. (2022) 35(2):135–40. 10.1055/s-0041-1742114 35237109 PMC8885156

[32] Dai S-X Gu H-X Gang W Tao Z Su H-J Zhan Y-L The value of CD8+CD28+/CD8+CD28-T cell balance in predicting concomitant gastrointestinal bleeding in patients with inflammatory bowel disease. J South Med Univ. (2016) 36(12):1609–15. 10.3969/j.issn.1673-4254.2016.12.04 27998853

[33] Xiang G Shanying L Sui P Hu P Che H. Clinical analysis of 250 inpatients with inflammatory bowel disease. Int Med Health Herald. (2005) (20):14–6.

[34] van Schaik FD Mooiweer E van der Have M Belderbos TD Ten Kate FJ Offerhaus GJ Adenomas in patients with inflammatory bowel disease are associated with an increased risk of advanced neoplasia. Inflamm Bowel Dis. (2013) 19(2):342–9. 10.1097/MIB.0b013e318286f771 23340679

[35] Thirlby RC Sobrino MA Randall JB. The long-term benefit of surgery on health-related quality of life in patients with inflammatory bowel disease. Arch Surg. (2001) 136(5):521–7. 10.1001/archsurg.136.5.521 11343542

[36] Lerang F Holst R Henriksen M Wåhlberg H Jelsness-Jørgensen LP. Antitumour necrosis factor alpha treatment in Crohn’s disease: long-term efficacy, side effects and need for surgery. Scand J Gastroenterol. (2022) 57(8):921–9. 10.1080/00365521.2022.2042592 35188443

[37] Zheng T-H Chen M Han S-Y Yang Y-X. New advances in the understanding of bone metabolism in inflammatory bowel disease. Chin J Pract Med. (2014) 41(11):85–7. 10.3760/cma.j.issn.1674-4756.2014.11.039

[38] Ballinger AB Savage MO Sanderson IR. Delayed puberty associated with inflammatory bowel disease. Pediatr Res. (2003) 53(2):205–10. 10.1203/01.PDR.0000047510.65483.C9 12538776

[39] Liu D-Y Zhao H-M. Notch-mTOR signalling-mediated immune response and energy metabolism axis: a possible key to preventing IBD relapse in Chinese medicine. World Chin J Digestol. (2016) 24:2617–24. 10.11569/wcjd.v24.i17.2617

